# Supplementary material for: Comparison of the ScreenFire and Xpert HPV assays for the detection of human papillomavirus and cervical precancer among women living with HIV in Malawi
Source: Infect Agent Cancer. 2024 May 17;19:24. doi: 10.1186/s13027-024-00585-4 (PMC11100048; doi:10.1186/s13027-024-00585-4)
Supplement: Supplementary file 1 — Additional file 1. [file 13027_2024_585_MOESM1_ESM.docx]

**Supplemental Table 1: Demographic and clinical characteristics of included participants**

|  | **HPV-positive on Self-collection by Xpert included in analysis,**  **N=279**  **n (%)** | **HPV-negative on Self-collection by Xpert included in analysis,**  **N=36**  **n (%)** |
| --- | --- | --- |
| **Age in years:** Median (range) | 36 (25-49) | 36 (25-47) |
| **Marital Status** | | |
| Married  Divorced/separated/widowed  Single | 171 (61.3%)  101 (36.2%)  7 (2.5%) | 25 (69.4%)  11 (30.6%)  0 (0%) |
| **Highest Education level attained** | | |
| None  Primary  Secondary or higher | 144 (51.6%)  100 (35.8%)  35 (12.5%) | 19 (52.8%)  14 (38.9%)  3 (8.3%) |
| **Monthly Income ^a^** | | |
| < 49,999 MK (< $47.76)  50,000 – 99,999 MK ($47.76-$95.53)  >100,000 MK (> $95.53) | 189 (67.7%)  53 (19.0%)  37 (13.3%) | 29 (80.6%)  4 (11.1%)  3 (8.3%) |
| **Occupation** | | |
| Self-employed^b^  Salaried employed  Unemployed /Piece Work | 133 (47.7%)  39 (14.0%)  107 (38.4%) | 20 (55.6%)  5 (13.9%)  11 (30.6%) |
| **Available Household Resources^c^** | | |
| Have Electricity | 83 (29.7%) | 8 (22.2%) |
| Have Running water | 94 (33.7%) | 9 (25.0%) |
| **Currently ART** | 262 (93.9%) | 32 (88.9%) |
| **History of smoking, n (%)** | 18 (6.5%) | 2 (5.6%) |
| **Prior cervical cancer screening** | 152 (54.5%) | 22 (61.1%) |
| **Gravidity:** Median (IQR) | 4 (3-5) | 3.5 (3-5) |
| **Number of lifetime sexual partners**  1  2-5  >5  Unknown | 28 (10.0%)  201 (72.0%)  46 (16.5%)  4 (1.4%) | 2 (5.6%)  28 (77.8%)  6 (16.7%)  0 (0%) |

^a^ MK = Malawian Kwacha. 1 MK = 0.00059 US Dollars as of 16/02/2024 (25,000 MK = 14.85 US Dollars; 100,000 MK = 59.42 US Dollars)

^b^ Non-salaried employment e.g. trader, vendor, sex-work, farming

^c^ Only those who answered yes are listed.

**Supplemental Table 2: Agreement between Xpert and ScreenFire on self-collected HPV specimens among 279 HIV-positive women in Malawi***

| **HPV**  **HPV Positivity** | **Xpert**  **self-collected HPV positive**  N (column %)  N=279** | **ScreenFire**  **self-collected result**  N (column %)  N=279** |
| --- | --- | --- |
| ***CIN2+*** | | |
| **Any HPV** | 49 (100%) | 47 (96%) |
| **HPV 16** | 15 (31%) | 15 (31%) |
| **HPV 18/45** | 7 (14%) | 6 (12%) |
| **HPV 31/33/52/58/35** | 34 (69%) | 25 (51%) |
| **Channels 4/5**^†^ | **Channel 4: HPV 51/59**  8 (16%) | **Channel 4:**  **HPV39/51/56/59/68**  11 (22%) |
|  | **Channel 5: HPV**  **39/56/66/68**  12 (24%) |  |
| **HPV Negative Participants** | 0 (0%) | 2 (4%) |
| ***<CIN2*** | | |
| **Any HPV** | 230 (100%) | 205 (89%) |
| **HPV 16** | 44 (19%) | 34 (15%) |
| **HPV 18/45** | 42 (18%) | 32 (14%) |
| **HPV 31/33/52/58/35** | 128 (56%) | 116 (50%) |
| **Channels 4/5**^†^ | **Channel 4: HPV 51/59**  44 (19%) | **Channel 4:**  **HPV39/51/56/59/68**  68 (30%) |
|  | **Channel 5: HPV**  **39/56/66/68**  53 (23%) |  |
| **HPV Negative Participants** | **0 (0%)** | 25 (11%) |

*Participants were HPV-positive on Xpert by self-collection at study entry.

*Participants can be HPV-positive for more than one channel; hence, the totals may add up to more than N=279. Self-collected specimens were tested with Xpert in July 2020 immediately after collection and their residual specimen divided into aliquot for freezer storage. The residual specimens were thawed and tested with ScreenFire between November 2022 and January 2023.

†Xpert Channel 4 (HPV 51/59) & Channel 5 (HPV 39/68/56/66) and ScreenFire Channel 4 (HPV 39/51/59/56/68) differ and cannot be directly compared.
